# Feasibility of diabetes self-management coaching program for individuals with type 2 diabetes in the Ethiopian primary care setting: a protocol for a feasibility mixed-methods parallel-group randomized controlled trial

**DOI:** 10.1186/s40814-024-01487-3

**Published:** 2024-04-08

**Authors:** Fikadu Ambaw Yehualashet, Dorothy Kessler, Segenet Bizuneh, Catherine Donnelly

**Affiliations:** 1 School of Rehabilitation Therapy, Faculty of Health Science, Queen’s University, 31 George St, Kingston, ON K7M 3N6 Canada; 2 College of Medicine and Health Sciences, The University of Gondar, Gondar, Ethiopia

**Keywords:** Feasibility, Diabetes, Self-management, Coaching, RCT, Primary care, Ethiopia

## Abstract

**Background:**

Diabetes mellitus is the third most prevalent chronic metabolic disorder and a significant contributor to disability and impaired quality of life globally. Diabetes self-management coaching is an emerging empowerment strategy for individuals with type 2 diabetes, enabling them to achieve their health and wellness goals. The current study aims to determine the feasibility of a diabetes self-management coaching program and its preliminary effectiveness on the clinical and psychosocial outcomes in the Ethiopian primary healthcare context.

**Methods:**

The study will employ a mixed-method feasibility randomized controlled trial design. Forty individuals with type 2 diabetes will be randomly allocated to treatment and control groups using block randomization. The primary feasibility outcomes include acceptability, eligibility, recruitment, and participant retention rates, which will be computed using descriptive analysis. The secondary outcomes are self-efficacy, self-care activity, quality of life, and glycated hemoglobin A1c. For normally distributed continuous variables, the mean difference within and between the groups will be determined by paired sample Student *t*-test and independent sample Student *t*-test, respectively. Non-parametric tests such as the Mann-Whitney *U* test, the Wilcoxon signed rank test, and the Friedman analysis of variance test will determine the median difference for variables that violated the normality assumption. A repeated measure analysis of variance will be considered to estimate the variance between the baseline, post-intervention, and post-follow-up measurements. A sample of 10 volunteers in the treatment group will participate in the qualitative interview to explore their experience with the diabetes self-management coaching program and overall feasibility. The study will follow a qualitative content analysis approach to analyze the qualitative data. Qualitative and quantitative findings will be integrated using a joint display technique.

**Discussion:**

Evidence reveals diabetes self-management coaching programs effectively improve HbA1c, self-efficacy, self-care activity, and quality of life. This study will determine the feasibility of a future large-scale randomized controlled trial on diabetes self-management coaching. The study will also provide evidence on the preliminary outcomes and contribute to improving the diabetes self-management experience and quality of life of individuals with type 2 diabetes.

**Trial registration:**

The trial was registered online at ClinicalTrials.gov on 12/04/2022 and received a unique registration number, NCT05336019, and the URL of the registry is https://beta.clinicaltrials.gov/study/NCT05336019.

**Supplementary Information:**

The online version contains supplementary material available at 10.1186/s40814-024-01487-3.

## Introduction

Diabetes is one of the leading global causes of morbidity and mortality [1]. The prevalence of diabetes is increasing at alarming rates,according to the [2] International Diabetes Federation (IDF) report, more than half a billion people live with diabetes globally, and this number will increase to 783.2 million by 2045 [3]. Approximately 80% of individuals with diabetes live in low- and middle-income countries (LMIC) [4, 5] and Ethiopia is one of the four African countries with the highest population of adults with diabetes [6]. According to IDF IDF [2], the IDF reported the prevalence of diabetes in Ethiopia is 3.3%. A systematic review and meta-analysis study in 2021 found a 6.5% pooled prevalence of diabetes in Ethiopia, ranging between 2% in the Tigray region and 14% in Dire Dawa [7]. Ethiopia does not have evidence-based national diabetes guidelines or standard referral criteria for diabetes management [8]. As a result, individuals with diabetes may receive substandard care. Notably, a systematic review and meta-analysis study revealed more than half of individuals with type 2 diabetes have poor self-care practices [9]. Furthermore, many people with type 2 diabetes in Ethiopia visit traditional healers and religious therapies like holy water to get a cure for diabetes mellitus [10] which makes self-management challenging.

An earlier cross-sectional study conducted in Addis Ababa, the capital of Ethiopia, showed that 87% of diabetic patients had regular clinical follow-up; however, 75% of participants required hospital admissions and did not receive diabetic education, and 95% of patients failed to monitor their blood glucose regularly [11]. A recent population-based cross-sectional study from Addis Ababa found that three-fourths of individuals with type 2 diabetes have glycated hemoglobin A1c (HbA1c) > 7.0% [12], indicating ongoing poor diabetes self-management. Furthermore, another hospital-based cross-sectional study in Southern Ethiopia revealed that 50% of individuals with diabetes suffered from one or more chronic complications: of these, 35%, 25%, and 15% acquire neuropathy, retinopathy, and nephropathy, respectively [13].

Although self-management is often used interchangeably with self-regulation, self-care, patient education, and patient counseling, it is beyond merely providing information and increasing awareness [14]. Self-management is recognized as tertiary prevention aiming to prevent the deterioration of health among individuals with chronic illness [14]. Self-management programs enable individuals to be active, responsible, informed, and autonomous in managing chronic illness’s physical, social, and emotional impact through collaboration with family, friends, and the healthcare provider(s) [15].

In recent years, Diabetes Self-Management (DSM) coaching, also called diabetes health coaching, has demonstrated a substantial effect on improving health [16]. The DSM coaching is a client-centered empowerment approach that enables individuals to self-manage diabetes (Radwan, [17]. It focuses on individual preferences, experiences, and values and engages participants in decision-making [18]. Studies in Taiwan, Indonesia, and Canada found that diabetes health coaching interventions can significantly improve HbA1c [19–21]. Systematic review and meta-analysis studies also demonstrate diabetes health coaching is an effective strategy to improve HbA1c [16, 17, 22, 23]. In addition, evidence suggests diabetes health coaching can lead to improvements in self-efficacy [24], self-care practice [19, 20], and quality of life [22, 25]. Despite the above evidence, culturally appropriate and effective DSM programs are lacking in most LMICs (Iregbu & Iregbu, [26], including Ethiopia.

In Ethiopia, an effective, patient-centered empowerment strategy to enhance diabetes self-management, build self-efficacy, improve quality of life, and control blood glucose is lacking, notably in the primary care setting. The DSM coaching program, which shows promising health outcomes in different settings, could be a practical approach in the primary care context of Ethiopia. Hence, the study will adapt a DSM coaching program from an evidence-based health coaching intervention [20] informed by an I-change model [27]. The study’s overarching goal is to determine the feasibility of implementing the DSM coaching program among individuals with type 2 diabetes in the primary care setting of Ethiopia and assess the program’s preliminary effectiveness in improving behavioral and clinical outcomes.

## Methods and materials of the study

### Primary objective

To determine the feasibility and acceptability of implementing an adapted DSM coaching program for individuals with type 2 diabetes in Ethiopia’s primary care settings.

### Secondary objective

To evaluate the potential effectiveness of the DSM coaching program on self-care activity, self-efficacy, quality of life, and HbA1c among individuals with type 2 diabetes.

### Phases of study

The study will have three phases: adaptation, implementation, and evaluation of the DSM coaching program.

#### Phase I: Adaptation of the DSM coaching intervention

This study will adapt an evidence-based intervention informed by the I-change model [20]. A purposively selected panel of experts will be invited to participate in adapting the DSM coaching program through the recommendation of selected departments. The Principal Investigator (PI) will ask the selected professionals to participate in the intervention adaptation process through a formal letter. A panel of multidisciplinary teams consisting of ten professionals and a patient representative will sit together to discuss and adapt the DSM coaching program and fidelity assessment tool at three-panel discussions.

The adaptation team will include one nurse, one internist (senior specialist doctor), two nurse researchers, two occupational therapists, two public health nutritionists, one physiotherapist, one epidemiologist (chronic disease researcher), and a patient representative.

The adaptation process will be iterative and will take 4 months. Panel members will review the candidate intervention manual before the panel discussion. The first panel discussion will focus on collecting feedback on the intervention component, reviewing each intervention component, and selecting potential intervention components for the DSM coaching program. A nominal group technique will be used to reach a consensus among panelists [28]. Accordingly, panelists will discuss thoroughly each intervention component based on the merits and demerits of incorporating it into the manual. Panel members will vote on the inclusion or exclusion of the intervention component and justify their decision. A consensus will be reached by a majority vote and supported by justification. The second panel discussion will emphasize revising selected intervention components and delivery methods. Two individuals with type 2 diabetes will participate in the adaptation process. During the third panel discussion, the panel members will review the draft DSM coaching intervention manual and approve the document. Furthermore, the adaptation process will use expert opinion and rating feedback to identify potential intervention components and delivery methods. The research team will prepare the content of the DSM coaching intervention manual by reviewing the literature and feedback from experts and the target population.

#### Phase II: Feasibility RCT

##### Study design

A convergent mixed-methods, single-blind feasibility randomized controlled trial (RCT) will be employed to assess the feasibility of the DSM coaching program. The study will adhere to the SPIRIT guideline for pilot and feasibility randomized trials [29]. The trial was registered online at ClinicalTrials.gov on 12/04/2022, and registration number NCT05336019 was received.

##### Study setting

The study will be conducted in the primary care settings of Gondar City, Amhara region, Ethiopia. Gondar City is about 740 km away from Addis Ababa, the capital of Ethiopia [30]. Christianity is the dominant religion, followed by Islam. The most common staple diet in Ethiopia is Injera, made up of teff flour after a consecutive date of fermentation. Almost all Ethiopians eat Injera with a stew of lentils or meat at least once daily [31]. People consume organic food items due to limited access to processed food items. Fasting is a common practice during Lent and Ramadan,hence, the study period will exclude these seasons. Gondar City has one referral hospital, nine primary care centers, and additional private health facilities.

According to unpublished Gondar City Health District reports, more than 10,000 individuals attend regular diabetes clinics in Gondar City health facilities. Individuals with diabetes received diabetes care at the referral hospital, private clinics, and primary care health centers. The hospital serves more than 2600 diabetes patients in the chronic disease clinic, and around 1312 patients have type 2 diabetes [32]. The hospital is a point of referral for newly diagnosed individuals with diabetes from health centers, individuals with complicated diabetes, and individuals with uncontrolled diabetes. Because of the absence of a district or a zonal hospital in the city, the city’s health system is inconsistent with the national referral system. As a result, the hospital provides primary, secondary, and tertiary care for individuals with diabetes. Most individuals with uncontrolled diabetes bypass the health centers and get care at the hospital. In addition, health centers also refer uncontrolled diabetes cases to the hospital. As a result, the study subjects will be recruited from the hospital. The research assistant will take informed consent from volunteers with type 2 diabetes who fulfill the screening criteria. Once the recruitment and baseline assessment are completed, the intervention will be administered in the selected health centers.

### Participant eligibility

#### Inclusion criteria

The study includes individuals who have been attending the diabetes clinic for at least 6 months, are living in Gondar City, are taking anti-diabetic medications, have a recent HbA1c ≥ 7% (within 3 months), and are between 18 and 65 years old.

#### Exclusion criteria

The study will exclude individuals with clinically confirmed mental illness, pregnancy [33], and cardio-vascular diabetes complications (neuropathy, nephropathy, retinopathy, stroke, gangrene, cancer, and cardiovascular disease). Individuals who are seriously ill and hospitalized during screening will also be excluded. Furthermore, as the DSM coaching program demands participants be physically active, individuals with physical impairment (visual deficit, hearing deficit) and lower extremity amputation or palsy will be excluded.

#### Sample size determination

A formal sample size calculation will not be applied for a feasibility RCT study [34]. Hertzog [35] suggests that a sample size of 10–40 participants per group is enough for feasibility studies (Hertzog, [35]. Hence, considering the study’s pilot nature, the intervention’s high intensity, and the limited research fund, we will recruit 40 participants. Twenty participants will be allocated to each arm of the study with a 1:1 ratio.

### Participant recruitment procedure

A 3-min audio record that briefly explains the purpose of the study and the recruitment process will be prepared in Amharic and played in the waiting area of the chronic disease clinic during working hours. In addition, a 1.5 m × 2 m poster describing the study objectives and the recruitment process will be prepared in Amharic and displayed in the waiting area. Nurses in the chronic disease clinic will receive a half-day training about the study, the screening material, and how to connect eligible individuals with the research assistant. Nurses working in the clinic will screen all individuals with type 2 diabetes and connect them with the research assistant. The research assistant will meet eligible individuals in a separate office in the clinic to discuss the study participation, information sheet, and informed consent.

### Randomization, allocation, and blinding

Participants will be assigned to the treatment and control groups using a block randomization technique with a block size of four [36]. An external researcher with no other role in the study will manually generate the random allocation sequence [37]. From a block of four, six possible combinations using treatment “T” and control “C” will be generated (TTCC, TCTC, TCCT, CCTT, CTTC, and CTCT). A nurse working outside the chronic disease clinic will draw the lottery 10 times from these six potential combinations to allocate 40 participants. The external researcher will document the sequence drawn by the nurse and prepare 40 opaque sealed envelopes to conceal the group allocation [37, 38]. The research assistant will receive the prepared envelopes, randomly allocate participants by opening the envelope in front of the participant, and inform participants of their allocation. All participants will be registered in the master linkage log sheet and informed of the next meeting date.

#### Blinding

Because of the nature of the intervention, it is impossible to mask study participants and the interventionist. However, the study assessors will be masked to the group allocation. To prevent accidental disclosure by study participants, the research assistant will provide a 5-min orientation for each participant just before each data collection period. To ensure the blinding of the assessors, three data collectors who are academic staff at the University of Gondar will be recruited for each assessment time (T1, T2, and T3). One data collector will be assigned for each phase and will not have contact with the study data and participants.

### Intervention arms of the study

**Fig 1:**
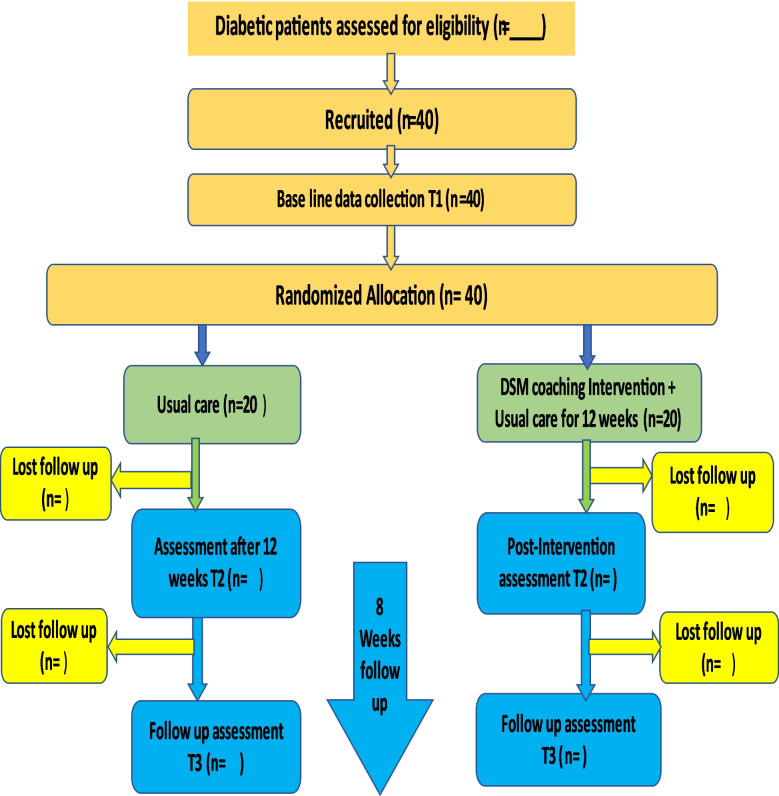
Study participant flow diagram based on the CONSORT guideline [29]. Key: *T1*, *T2*, *T3* represent time of assessment, *n* number

The study will have two arms: the treatment group will receive the DSM coaching program, and the control group will continue receiving the usual care (see Fig. 1).

*Treatment group:* The treatment group will attend a 12-week DSM coaching program culturally adapted from the I-change model [27], an evidence-based health coaching intervention [20]. The DSM coaching program will have six interactive group sessions, including an overview of diabetes, goal setting, dietary management, exercise, blood glucose monitoring and medication, and foot care. In addition, the DSM coaching will also include four individual home-based coaching sessions. Furthermore, a family member who is a direct care provider will have a 10-min orientation after every individual home-based coaching session with the participants.

The principal investigator, who has completed a coaching and counseling course at Queen’s University, Kingston, Canada, will facilitate the DSM coaching sessions. The coach will deliver six 2-h group coaching sessions every 2 weeks for 3 months. The coach will use interactive discussions, experience sharing, demonstration, and home-take assignments to address the program goals. The intervention group will also continue receiving the usual care in the clinic (see Additional file 1: the DSM coaching program).

#### Control group

The control group will continue receiving the usual care in the chronic disease clinic. The usual care is biweekly or monthly clinical appointments, depending on the severity of the illness. Services provided during these clinical visits include history taking, physical examination, laboratory investigation, and medication refills. The available treatments for type 2 diabetes may include oral hypoglycemic agents such as metformin, glipizide, and nateglinide, and injectable insulin. Depending on the severity of the illness and the blood glucose level, doctors may prescribe an oral hypoglycemic agent, insulin, or a combination of both.

### Outcome measures

#### Primary outcomes

##### Feasibility outcomes

The current study will address the eligibility rate, recruitment rate, retention rates, adherence rate, and the acceptability of the DSM coaching program. The progression criteria will determine the success of the DSM coaching program (Table 1).

**Table 1:** Progression criteria for the feasibility of the DSM coaching program

**Feasibility outcomes**	**Operational definitions**	**Progression criteria/success**	**Source of data**
Eligibility rate	It is the proportion of people who fulfill the screening criteria.	$\ge$ 50% will be acceptable for the future trial	Screening records
Recruitment capacity (rate)	It is the proportion of eligible individuals willing to give consent and randomized to the study in 2-month period.	$\ge$ 80% (32+) recruitment rate.	Record review
Intervention adherence	An intervention adherence is the participant’s compliance with attending all group sessions, and all home-based coaching sessions.	$\ge$ 80% adherence rate	Session attendances of individual and group sessions.
Retention	The ability of the program to retain participants in the study. The proportion of study participants who completed the study and evaluated at the end of the intervention T2 and end of follow-up T3.	$\ge$ 80% retention rate	Record review
Acceptability	It is the perception of study participants that a given intervention is agreeable, palatable, or satisfactory.	$\ge$ 75% TAAS score	TAAS survey
Coaching session fidelity	It is the proportion of individual coaching sessions rated as moderate or high.	$\ge$ 80% will be an acceptable level of fidelity.	Coaching sessions fidelity checklist rated by an OT
DSM coaching Program fidelity	The proportion of fidelity assessment for the DSM coaching program	$\ge$ 80% will be an acceptable level of fidelity.	DSM coaching program fidelity assessment checklist

##### Eligibility rate

The eligibility rate is the proportion of individuals who fulfill inclusion criteria among those screened. It will be calculated as Eligibility rate = (NE/NS) × 100, where NE is the number of eligible participants, and NS is the number of participants screened.

##### Recruitment rate

The recruitment rate is the proportion of people enrolled (randomized) in the program. It will be computed as Recruitment rate = (NR/NE) × 100, where NR is the number of randomized participants, and NE is the number of eligible participants.

##### Retention rate

The retention rate is the proportion of participants who complete the intervention. The retention rates can be computed as Retention rate = (NA/NR) × 100, where NA is the number of participants assessed at T3, and NR is the number of randomized participants.

##### Adherence rate

The adherence rate is the proportion of participants who attended 80% of the DSM coaching (group + individual) sessions. Adherence rate will be calculated as Adherence rate = (NA/NP) × 100, where NA is the number of participants who attended 80% of the sessions, and NP is the total number of participants.

##### Acceptability

Acceptability determines how well the target population receives a program and how it meets the needs of the target population [39]. Acceptability of the DSM coaching program will be assessed using the Treatment Acceptability/Adherence Scale (TAAS) [40]. The TAAS is a 10-item Likert scale with a value ranging between 1 and 7.

##### Fidelity of the DSM coaching program

The fidelity of the DSM coaching program will be assessed using the Comprehensive Intervention Fidelity Guide (CIFG) designed by Gearing. The CIFG is a guide with four core elements: design, training, delivery, and receipt fidelity proposed to examine an overall intervention fidelity [41]. The DSM Coaching Fidelity Measure (DSMC-FM) will assess the fidelity of the DSM coaching sessions. The DSMC-FM is informed by the Occupational Performance Coaching-Fidelity Measure (OPC-FM) [42]. The DSMC-FM has 23 items, of which 16 are critical components that focus on the coach’s behavior and practices. Four items address the client’s responses and behavior,the rest (3 are distinguished items needing improvement. An occupational therapist will review the recordings of the DSM coaching individual sessions and assess the fidelity of the coaching sessions.

### Secondary outcomes

The secondary outcomes of the study encompass self-efficacy, self-care activity, quality of life, and HbA1c. These outcomes will be assessed at baseline (T1), post-intervention (T2), and post-follow-up period (T3) (Table 2).

- A. Self-care activity
  The diabetes self-care activity is the ability of an individual with type 2 diabetes to follow a healthy eating plan, perform regular exercise, monitor blood glucose, perform foot care, adhere to diabetes medication/s, and cease cigarette smoking [43]. Self-care activity will be measured using the Summary of Diabetes Self-Care Activity tool (SDSCA) [43]. The tool will be culturally translated and validated for face and content validity by a panel of experts.
- B. Self-efficacy
  Self-efficacy is an individual’s belief and confidence in their ability to perform intended activities that affect their life and control over how these activities are experienced [44, 45]. Self-efficacy will be measured using the Stanford Self-Management Resource Center (SMRC) diabetes self-efficacy scale, which has an eight-item Likert Scale [46]. The tool will be translated into the local language, Amharic, and undergo content validation by a panel of experts.
- C. Quality of life
  As defined by the WHO, quality of life is an individual’s perception of their position in life in the context of the culture and value systems in which they live and their goals, expectations, standards, and concerns [47]. The quality of life of individuals with type 2 diabetes will be assessed by a valid and reliable WHOQOL-BREF tool [48]. The tool has demonstrated reliability and construct validity [48]. The WHOQOL-BREF tool has 26 items addressing the physical, psychological, social, and environmental health domains with five ordinal scales [48].
- D. Glycated hemoglobin A1C
  HbA1C is a reliable measure of long-term glucose monitoring recommended by the WHO and the American Diabetes Association (ADA) [49]. HbA1c < 7% is considered good glycemic control, and HbA1c equal to or greater than 7% will be regarded as an uncontrolled glucose level [50].

**Table 2:** Outcome assessment timeline showing list of assessment items (outcomes) and corresponding evaluation time

**Outcome variables**	**Assessment time in weeks**												
	T1–W0	W1–3	W4	W5	W6	W7	W8	W9	W10	W11	W12	T2–W13	T3–W24
**Acceptability**													﻿√
Eligibility rate	√												
**Recruitment**	√												
Retention rate	√										√		√
**Adherence rate**		√	√	√	√	√	√	√	√	√	√		
TAAS												√	
**Fidelity-CIFG**	√	√	√		√		√		√		√		
DSMC-FM				√		√		√		√			
**Self-efficacy**	√											√	√
Self-care practice	√											√	√
**Quality of life**	√											√	√
HbA1c	√											√	√
**Blood pressure**	√											√	√
Body mass index	√											√	√

*T1* baseline, *T2* post-intervention assessment, *T3* post-follow-up assessment, *W* week

#### Data collection

Data on participants’ demographic, behavioral, and clinical characteristics will be collected through interviews and reviewing patient medical records. Clinical characteristics such as HbA1c and blood pressure will be retrieved using a chart review form. The weight and height of participants will be measured during their clinical visit to compute body mass index.

Data on self-care activities, self-efficacy, and quality-of-life will be collected using an interviewer-administered data collection technique at baseline (T1), at the end of the intervention (T2), and at the end of the follow-up period (T3). All three data collection periods will be scheduled at least 2 weeks after the holidays as the dietary practice during holidays affects study outcomes. Three MSc nurse data collectors will receive a 1-day training on questionnaire administration procedures, the study instruments, study blinding, and ethical issues related to data collection. Each data collector will work only once to keep the data collectors masked about group allocation. Additionally, all study participants will attend a 5-min orientation to ensure they do not disclose their group to the data collector during the interview. Data related to the quantitative feasibility outcomes: eligibility, recruitment, adherence, and retention rate, will be collected from the recruitment documents, session attendance sheets, and assessment reports throughout the study process. The TAAS assessment will be carried out along with post-follow-up data collection. However, only the treatment group participants will be asked about the program’s acceptability.

### Study participant retention

Retaining adequate study participants is challenging for many interventional studies involving human subjects. Studies suggest different strategies to increase the retention rate [51, 52]. One of the strategies to retain participants in this study is building effective relationships and treating participants respectfully and compassionately. Hence, the research assistants and data collectors will demonstrate respect and compassion for the study participants. A close follow-up throughout the study period will be the other mechanism to ensure participant retention. The research assistant will closely follow participants through regular attendance and make reminder calls before each session. The research assistant, principal investigator, and participants will discuss and arrange meeting schedules for the group coaching and individual home-based coaching sessions. Participant transport costs will be covered to encourage their participation. In addition, with tangible evidence, any cost incurred by the participant for the purpose of the study will be refunded. The group session will be designed to create active participation through discussion, role play, and demonstration, which will make the sessions attractive and enhance participation.

### Data analysis and management

An independent statistician will enter, code, and clean the data using Epi-Info version 7.3.2. The data will be imported to SPSS version 29 for analysis**.** The principal investigator will analyze the data in consultation with statisticians and the research team. An intention-to-treat (ITT) analysis technique will be followed to analyze the quantitative data [53]. An ITT analysis will run data of all participants irrespective of their adherence to the program [54], and missing values will be filled using multiple imputation techniques [55]. Baseline differences between the treatment and control groups will be examined using *t*-tests. Frequencies, percentages, mean, median, standard deviation, and interquartile range will be computed to describe the population. A *p*-value < 0.05% with a 95% confidence level will be used to determine statistical significance.

Differences in the effect size of the secondary outcomes of the study will be computed following a test of assumptions. For normally distributed continuous outcomes, the mean difference within and between the groups will be determined by paired sample Student *t*-test and independent sample Student *t*-test, respectively. Non-parametric tests such as the Mann–Whitney *U* test, Wilcoxon signed rank test, and Friedman analysis of variance test will be computed to determine the median difference for variables that violate the normality assumption. A repeated measure analysis of variance will be considered to estimate the variance between the baseline, post-intervention, and post-follow-up measurements. If a difference is detected between the two groups, a covariate analysis approach or multivariate analysis technique will be considered to rule out the effect of covariates using the pretest score and age of the participant as the covariate.

#### Phase III: Qualitative study

##### *Study design*

A qualitative description [56] approach will be followed to examine the acceptability of the DSM coaching program. This approach allows the researcher to explore participants’ perspectives and understand the barriers and facilitators of a given phenomenon [56, 57]. Hence, the study will explore the acceptability of the DSM coaching program, including the participants’ perspectives on the program, the challenges, and the enablers of implementing the program in the primary care context.

##### Sampling procedure and sample size

A purposive sampling method with a maximum variation technique [58] will be used to include participants of different genders, duration of diabetes, educational status, and program adherence level. The study will recruit ten individuals with type 2 diabetes who participated in the DSM coaching program.

##### *Recruitment of participants*

At the end of the group DSM coaching session, the principal investigator and the research assistant will invite eligible participants to participate in the in-depth interview.

##### *Data collection*

An experienced qualitative researcher will conduct the interviews. Study participants will sign an informed consent form and have the option to decide on the recording of the interview before the start of the session. A semi-structured interview question addressing the acceptability of the DSM coaching program will be prepared in Amharic to explore the experience of individuals with the DSM coaching program. Interviews will be audio-recorded, transcribed verbatim by a transcriber, and translated into English for analysis.

##### Qualitative data analysis and management

Data will be analyzed using qualitative content analysis [56]. An experienced transcriber will transcribe all the interview records in Amharic. Two of these transcripts will be translated into English by a professional translator and used by the research team to prepare a codebook. The data analysis will follow a three-step inductive content analysis approach: preparation, organization, and reporting of the analysis process [59]. The preparation phase includes selecting the unit of analysis and making sense of the data. The organization phase includes open coding, grouping codes, categorizing, and abstraction. The third step is reporting the findings using a model or conceptual system. NVivo software version 14 will be used to code, categorize, and recategorize the qualitative data [60].

##### *Data integration*

**Fig 2:**
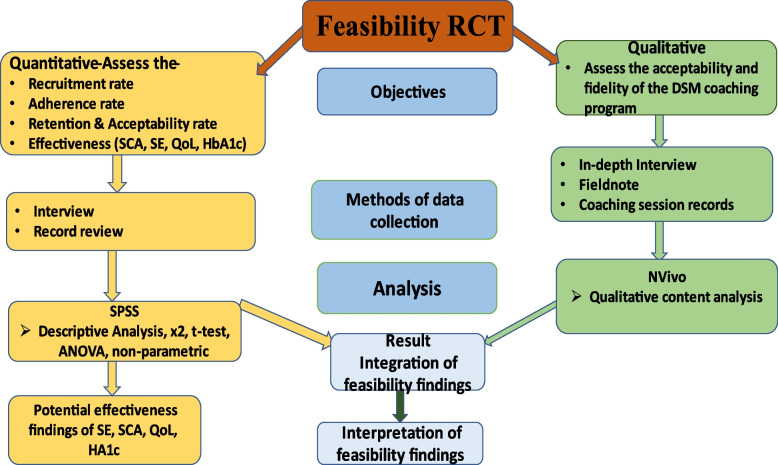
A flow diagram showing the mixed-method data integration process of the DSM coaching program

The qualitative and quantitative findings will be integrated at the resulting level [61] and interpreted to answer the feasibility research questions. Data will be presented in a joint display technique to interpret findings (Fig. 2).

## Discussion

Self-management strategies rely on individuals’ preferences, build on prior knowledge, recognize contextual factors, and encourage active engagement in chronic illness care [62]. Establishing collaboration between patients, health care providers, family members, and the community at the primary care level is a means for successful self-management of chronic illness [63]. Diabetes self-management in the primary care of Ethiopia is problematic due to poor linkage within the health systems, poor quality of services, and lack of public awareness. Individuals with diabetes may live with the illness for many years and are supposed to manage a significant portion of the self-care activities such as diet, exercise, medication, foot care, and blood glucose monitoring by themselves [63].

In resource-limited settings where community support programs and linkage are lacking, primary care agencies need to think of alternative schemes to address the self-management needs of individuals with diabetes. The practice of self-management strategies like goal setting in the primary care settings is challenged by patient readiness, skill, and attitude, provider’s attitude and skill, and lack of time [64]. Diabetes self-management coaching has shown significant improvement in self-efficacy, self-care behavior, quality of life, and clinical parameters among individuals with type 2 diabetes in developed countries. However, one of the challenges in adapting and tailoring a complex health intervention in resource-limited countries is the program’s acceptability and implementation feasibility. Hence, the current study will explore the acceptability of the DSM coaching program. Furthermore, the study will evaluate the preliminary effectiveness of the DSM coaching program on self-efficacy, self-care activity, quality of life, and HbA1c. Evidence generated from this feasibility/pilot study will be used to design and implement a definitive RCT among individuals with type 2 diabetes.

### Strengths and limitations of the study

The study will introduce a culturally adapted and contextually tailored DSM coaching program through an iterative process supported by a supervisory committee and a panel of multidisciplinary experts. In addition, the study will be the first to introduce coaching as an intervention in the Ethiopian primary care context; hence, it will be a foundation for future definitive trials in the area. Furthermore, the preliminary outcomes of the study will be used to design DSM coaching programs in the context of low-income countries. Due to the nature of the study, the trial implementor, research assistant, and study participants will not be blinded regarding group allocation. The short follow-up period of the study might make it challenging to make a meaningful conclusion on the long-term behavioral outcomes. Furthermore, the lack of adequate information about the amount, type, and effect of processed diets on diabetes management and the effect of religious and cultural practices on diabetes management limits our ability to characterize the study population.

### Supplementary Information

**Additional file 1**: The DSM coaching program.

## Data Availability

All required data will be available upon request; please contact the principal investigator.

## Abbreviations

ADA: American Diabetes Association
ANOVA: Analysis of Variance
CIFG: Comprehensive Intervention Fidelity Guide
DSM: Diabetes Self-Management
DSMC-FM: Diabetes Self-Management Coaching Fidelity Measure
HbA1c: Glycated Hemoglobin A1c
IDF: International Diabetes Federation
ITT: Intention to Treat
LMIC: Low- and Middle- Income Countries
WHO: World Health Organization
OPC-FM: Occupational Performance Coaching-Fidelity Measure
RCT: Randomized Controlled Trial
SDSCA: Summary of Diabetes Self-Care Activity
SMRC: Stanford Self-Management Resource Center
SPIRIT: Standard Protocol Item: Recommendations for Interventional Trials
SPSS: Statistical Package for the Social Sciences
TAAS: Treatment Acceptability/Adherence Scale
UGCSH: University of Gondar Comprehensive Specialized Hospital
WHO: World Health Organization
WHO-QoL: World Health Organization-Quality of life
