# Supplementary material for: Feasibility of diabetes self-management coaching program for individuals with type 2 diabetes in the Ethiopian primary care setting: a protocol for a feasibility mixed-methods parallel-group randomized controlled trial
Source: Pilot Feasibility Stud. 2024 Apr 8;10:59. doi: 10.1186/s40814-024-01487-3 (PMC11000297; doi:10.1186/s40814-024-01487-3)
Supplement: Supplementary file 1 — Additional file 1: The DSM coaching program. [file 40814_2024_1487_MOESM1_ESM.docx]

**Annex 2:** **DSM Coaching program group and individual coaching sessions schedule**

| **Weeks** | **Type of Sessions** | **Delivery method/activities** | **Agenda of coaching session** | **Facilitator** | **Duration** |
| --- | --- | --- | --- | --- | --- |
| Week 1 &2 | Group session 1 | Group discussion | **Reflection, experience sharing, and Basics of diabetes** | Coach and Coachees | 2 Hours |
|  |  |  | - Participant introduction |  | 10’ |
|  |  |  | - Experience sharing |  | 10’ |
|  |  |  | - Reflection |  | 10’ |
|  |  |  | ***Health break*** |  | 10’ |
|  |  |  | - Basics of diabetes |  | 40’ |
|  |  |  | - Diabetes complications |  | 35’ |
| Week 3 & 4 | Group Session 2 | - Group discussion - Group exercise - Individual activity | **Goal setting** | Coach and Coachees | 2 hours |
|  |  |  | - Recap of previous sessions |  | 10’ |
|  |  |  | - Definition of goal setting |  | 40’ |
|  |  |  | ***Health break*** |  | 10’ |
|  |  |  | - How can we set goals |  | 25’ |
|  |  |  | - Quality of personal goals |  | 30’ |
|  |  |  | - Home take assignment orientation |  | 5’ |
| Week 5 | Group Session 3 | - Group discussion - Group exercise - Home take activities | **Diet management** | Coach and Coachees | 2 hours |
|  |  |  | - Recap previous session |  | 10’ |
|  |  |  | - Importance of diet for diabetes and barriers |  | 50’ |
|  |  |  | ***Health break*** |  | 10’ |
|  |  |  | - Dietary recommendations for diabetes |  | 45’ |
|  |  |  | - Home take assignment orientation |  | 5’ |
| Week 6 | Individual Coaching session 1 | - One-on-one coaching - Family orientation | **Individual home-based dietary management coaching** | Coach,  Coaches & family | 40 min |
|  |  |  | - Introduction and recap of the previous session |  | 5’ |
|  |  |  | - Undertake coaching session |  | 25’ |
|  |  |  | - Family orientation on diet management |  | 10’ |
|  |  |  | - Home take assignment on preparing and implementing diet plan for 2 weeks |  |  |
| Week 7 | Group Session 4 | - Group discussion - Experience sharing - Home take activities | **Regular physical exercise** | Coach and Coachees |  |
|  |  |  | - Recap previous session |  | 10’ |
|  |  |  | - Role of exercise in diabetes |  | 55’ |
|  |  |  | ***Health break*** |  | 10 |
|  |  |  | - Exercise recommendations for diabetics |  | 40 |
|  |  |  | - Home take assignment orientation |  | 5’ |
| Week 8 | Individual Coaching session 2 | - One-on-one coaching - Family orientation | **Individual home-based session on physical exercise** | Coach and Coachee | 40 min |
|  |  |  | - Introduction and recap of the previous session |  | 5’ |
|  |  |  | - Undertake exercise coaching session |  | 25’ |
|  |  |  | - Family orientation on regular exercise | Coach and family member | 10’ |
|  |  |  | - Home take assignment on regular exercise | Coachee |  |
| Week 9 | Group Session 5 | - Demonstration - Group discussion - Home take assignment | **Glucose monitoring and medication compliance** | Coach and Coachees | 2 hours |
|  |  |  | - Recap previous session |  | 10’ |
|  |  |  | - Blood glucose monitoring |  | 25’ |
|  |  |  | - Importance of measuring blood glucose |  | 25’ |
|  |  |  | ***Health break*** |  | 10 |
|  |  |  | - Role of medication for diabetics |  | 25’ |
|  |  |  | - Medications compliance |  | 20’ |
|  |  |  | - Home take assignments |  | 5’ |
| Week 10 | Individual Coaching session 3 | - One-on-one coaching - Family orientation | - **Individual coaching on BGM and medication adherence** | Coach and Coachee | 45 min |
|  |  |  | - Introduction and recap of the previous session |  | 5’ |
|  |  |  | - Undertake coaching session |  | 30’ |
|  |  |  | - Family orientation on blood glucose monitoring and medication | Coach and family member | 10’ |
|  |  |  | - Home take assignment on preparing and implementing glucose monitoring and medication adherence | Coachee |  |
| Week 11 | Group session 6 | - Group discussion - Demonstration - Home take assignment | **Foot care** | Coach and Coachees | 2 hours |
|  |  |  | - Recap previous session |  | 10’ |
|  |  |  | - Diabetics foot care |  | 50’ |
|  |  |  | ***Health break*** |  | 10’ |
|  |  |  | - Foot care recommendations |  | 45’ |
|  |  |  | - Home take assignment |  | 5’ |
| Week 12 | Individual Coaching session 4 | - One-on-one coaching - Family orientation | - **Individual home-based session on foot care** | Coach and Coachee | 40 min |
|  |  |  | - Introduction and recap of the previous session |  | 5’ |
|  |  |  | - Undertake coaching session |  | 25’ |
|  |  |  | - Family orientation on foot care | Coach and family member | 10’ |
|  |  |  | - Home take assignment on preparing and implementing foot care plan | Coachee |  |
